# Supplementary material for: The Scalable Brain Atlas: Instant Web-Based Access to Public Brain Atlases and Related Content
Source: Neuroinformatics. 2015 Feb 17;13(3):353–66. doi: 10.1007/s12021-014-9258-x (PMC4469098; doi:10.1007/s12021-014-9258-x)
Supplement: Supplementary file 1 — Appendix 1: description of JSON files that make up an atlas template (DOCX 25 kb) [file 12021_2014_9258_MOESM1_ESM.docx]

# Appendix A. Scalable Brain Atlas – template data storage

The latest version if this text is available at <http://scalablebrainatlas.incf.org/docs/template_data.php>.

The SBA stores atlas template data as a set of plain text files, each containing a data object in Javascript Object Notation (JSON). The data for template X are stored in the folder <http://scalablebrainatlas.incf.org/X/template>, valid values of X are listed here: <http://scalablebrainatlas.incf.org/services/listtemplates.php>.

Each template folder contains the following files (version 2):

- **config.json** – metadata and configuration parameters, as specified in the next section.
- **rgb2acr.json** – contains a mapping from each region-ID to the region’s acronym. The region-ID is encoded as a Red-Green-Blue (RGB) value with two hexadecimal values for each channel.
  Acronyms do not need to have a region-ID in these cases:
  1. The acronym is part of the template but no shape data is provided
  2. The acronym represents a superstructure that has no shape data of its own.
- **acr2full.json** – contains a mapping from each brain region acronym (abbreviation) to its full name.
  If no full name is defined for an acronym, its region-ID is used instead.
- **acr2parent.json** – contains a mapping from a brain region acronym to its parent acronym, to construct a hierarchy (optional).
- **alias2acr.json** – contains a mapping from alternative brain region names (aliases) to the standard acronym (optional).
- **svgpaths.json** – contains a list of all shape data, encoded as SVG-paths, in the template (i.e. for all regions in all slices). The list index is called *path-ID*.
- **brainslices.json** – contains for each slice-ID a list that contains, for each region-ID that is visible in the slice, a list of path-IDs that make up the region.
- **brainregions.json** – contains for each region-ID a list that contains, for each slice-ID that contains the region, a list of path-IDs that make up the region.
- **hulls.json** – contains for each slice-ID an SVG-path that represents its convex hull.
- **slicepos.json** – contains the position of each slice in millimeters from the reference point defined in config.json
- **origslicepos.json –** same as slicepos.json, but now for each slice in the original data set.
- **rgbvolumes.json** – the volume of each region in cubic millimeters
- **rgbcenters.json** – the centerpoint of each region in stereotactic coordinates. The centerpoint is obtained by peeling of the region until a single pixel remains. This procedure ensures that the centerpoint lies inside the region (unlike the center of gravity).

In the above, slice-ID refers to the SBA internal coronal slice numbering, which starts at 0 for the most anterior slice. SBA downsamples coronal slices to arrive at a total of 100-150 slices.

The text for the About section of template X is stored in the file <http://scalablebrainatlas.incf.org/X/creditsLinks.snip>

## Configuration parameters

The file **config.json** contains the following parameters (version 2):

- **version** – the version number of the config file
- **release** – the maturity of the template, either “alpha”, “beta”, or “stable”
- **species** – the animal species
- **ncbiName** – the species name according to the ncbi taxonomy database <http://www.ncbi.nlm.nih.gov/taxonomy>.
- **ncbiId** – the species ID of the NCBI taxonomy database.
- **nlxName** – the species name according to the Neuroscience lexicon <http://neurolex.org>
- **nlxId** – the species ID as used by NeuroLex.
- **templateName** –full name of the template
- **boundingBox** – bounding box in SVG coordinates of a coronal slice, the same bounding box is used for all slices. Bounding box contains four numbers: Left, Bottom, Width, Height.
- **whiteMatterAcronyms** – list of acronyms that should get a lighter color in the slice viewer.

Example: [“cc”]

- **slicePositionUnit** – the units of the posterior-anterior slice positions stored in *slicepos.json*
- **slicePositionDescription** – the description of the slice positions stored in slicepos.json. Example: “anterior distance to center of anterior commissure”
- **sliceUnits** – the units of the left-right and bottom-top dimensions of a coronal slice.
  Example: [“mm”,”mm”]
- **sliceCoordFrame** –defines a rectangular coordinate frame in SVG coordinates of a coronal slice. Uses same format as *BoundingBox*. Used in conjunction with *sliceXLim* and *sliceYLim*
- **sliceXLim** – defines, for the rectangle defined by *sliceCoordFrame*, what the physical coordinates of its left and right borders are. Example: [-8,8]
- **sliceYLim** – defines, for the rectangle defined by *sliceCoordFrame*, what the physical coordinates of its bottom and top borders are. Example: [-8,8]
- **sliceCoordSystem** – if not defined, slices are considered to be in anterior view, whereby the left side of each slice represents the right hemisphere of the brain (as seen by the subject). If set to "RAS", slices are considered to be in posterior view. RAS means that the X, Y and Z coordinate represent the Left to **R**ight, Posterior to **A**nterior and Inferior to Superior axis, respectively.
- **sliceRange** – defines how the original slices were resampled by the SBA, as a triplet of (1) start slice, (2) end slice, and (3) step size. Example: [1341,84,-10]. The internal SBA number starts at 0 for the most anterior slice.
- **plugins** – list of plugins that are compatible with this template. Default plugins are defined in the default configuration file <http://scalablebrainatlas.incf.org/main/config.json>
- **overlays** – contains for each overlay modality a list with the following parameters:
  - **source** – the file location of the coronal slice images as a *printf* format string, with a substitution field for the original slice number. Example: “coronal_GRE/p80_average_gre_%03d.jpg”
  - **anchorUnit** – specifies the units of the *anchorBox* field below. Supports only the value “SVG”.
  - **anchorBox** – the coordinate frame to place the image in. Uses same format as *BoundingBox*. If not specified, the overlay image is assumed to lie within the *sliceCoordFrame*.
